# Secondary Metabolites of *Fomitopsis betulina*: Chemical Structures, Biological Activity and Application Prospects

**DOI:** 10.3390/jof10090616

**Published:** 2024-08-29

**Authors:** Jianghao Li, Ziheng Li, Yingce Duan, Chengwei Liu, Meixia Yan

**Affiliations:** 1 Key Laboratory for Enzyme and Enzyme-Like Material Engineering of Heilongjiang, College of Life Science, Northeast Forestry University, Harbin 150040, China; jiangchen921@163.com (J.L.); a17856112016@163.com (Z.L.); yingceduan@163.com (Y.D.); 2 Institute of Special Animal and Plant Sciences, Chinese Academy of Agricultural Sciences, Changchun 130112, China

**Keywords:** *Fomitopsis betulina*, secondary metabolites, cultivation, terpenoids, biosynthesis

## Abstract

*Fomitopsis betulina*, as a macrofungus with both medicinal and dietary applications, is renowned for its rich content of bioactive substances. The recent advancements in research have significantly enhanced our understanding of its polysaccharides, cellulose-degrading enzymes, and wide range of secondary metabolites. This paper provides a comprehensive review of the artificial cultivation techniques and the chemical profiling of over 100 secondary metabolites identified in *F. betulina*, including terpenoids, phenols, and various other classes. These compounds exhibit notable pharmacological activities, such as anti-cancer, anti-inflammatory, antimicrobial, antiviral, and anti-malarial effects. Moreover, this review delves into the genomic analysis of *F. betulina*, focusing on the prediction and classification of terpene synthases, which play a crucial role in the biosynthesis of these bioactive compounds. This insight is instrumental for potentially facilitating future biochemical studies and pharmaceutical applications. Through this review, we aim to solidify the foundation for future in-depth studies and the development of new drugs derived from this promising natural resource.

## 1. Introduction

In the burgeoning field of medicinal mycology, the exploration of fungi as a source of novel bioactive compounds has gained considerable attention. Well-known medicinal fungi such as *Ganoderma lucidum* [1], *Hericium erinaceus* [2], *Agrocybe aegerita* [3] and *Laetiporus* species [4] exemplify this trend. Fungi, particularly large polypores like *Fomitopsis betulina*, are renowned for their diverse and potent pharmacological properties, which make them valuable in traditional and modern medicine alike. Biologically active compounds typically consist of multiple chemical components, often polysaccharides or terpenoids [5]. *F. betulina,* previously classified as *Piptoporus betulinus*, belongs to the Basidiomycota phylum, within the Agaricomycetes class, Polyporaceae family, and Fomitopsidaceae genus. This saprophytic fungus predominantly grows on trees belonging to the Betula genus. It synthesizes cellulase and hemicellulase enzymes that effectively break down cellulose. Notably, the fungus is edible in its juvenile stages [6]. It is widely distributed across Europe, North America, and the northern regions of Asia [7]. The fungal body is characterized by its white flesh and a leathery outer skin. The successful laboratory cultivation of *F. betulina*’s fruiting bodies has been documented, facilitating further research into its properties and potential applications [8].

Human utilization of *F. betulina* can be traced back to thousands of years ago. Notably, in 1991, the discovery of a 5000-year-old ice mummy in Italy, which was found with two specimens of *F. betulina*, suggests that ancient humans may have recognized and utilized its medicinal properties. Scholars hypothesize that these early people exploited *F. betulina* for its potential therapeutic effects, possibly using it to treat intestinal parasites or as an antibacterial agent [9]. Although there is some debate regarding the intended uses of the specimens found with the ice mummy, the enduring presence of *F. betulina* in cultural practices underscores its significance. For instance, Russian folklore describes the use of *F. betulina* fruiting bodies brewed as mushroom tea to alleviate fatigue and enhance immune function [10]. Additionally, in regions such as Siberia, the Baltic states, and Finland, this birch polypore fungus is commonly used to treat various types of cancer [11]. In contemporary times, driven by advances in technology and increased research capabilities, it has been established that *F. betulina* contains compounds with a spectrum of biological activities, including antibacterial, anti-parasitic, antiviral, anti-inflammatory, anti-cancer, and immunomodulatory effects [12].

Despite its promising therapeutic potential, the cultivation of *F. betulina* is challenging, which has historically limited its study and application. However, the recent advances in cultivation techniques have begun to unlock the possibility of studying these fungi under controlled conditions, thereby enhancing the reliability of compound extraction and allowing for consistent study results. This paper reviews the current state of knowledge regarding the cultivation, chemical composition, medicinal utility, genomic information, and predicted terpene biosynthetic gene of *F. betulina*, setting the stage for future research that could lead to new pharmacological developments. Through this exploration, we aim to contribute to a deeper understanding of its role in medicine and its potential integration into therapeutic applications.

## 2. Artificial Cultivation of *F. betulina*

Wild *F. betulina* is commonly found on the trunks of birch trees. Although it has a medicinal history spanning thousands of years, it is only within the last few decades that methodologies for its artificial cultivation have been systematically developed. In 2016, Pleszczyńska, M. et al. [13] pioneered a method utilizing fresh birch sawdust enriched with mineral additives (ranging from 15% to 45%) and organic supplements as a growth substrate for *F. betulina.* They explored substrate moisture contents between 45% and 65% and inoculated the substrates with four distinct strains of *F. betulina*. Their experiments indicated no significant correlation between the duration of mycelial colonization and the variations in organic supplements or substrate moisture levels. Intriguingly, the study identified that the strain-specific responses were the sole determinant influencing successful colonization; specifically, strain PB01 exhibited the induction of cold shock after 28 days of cultivation, followed by the development of primordia within 8–10 days, which then matured into fruiting bodies. Further analysis revealed that while the presence of mycelia did not correlate significantly with the moisture content or the organic supplementation of the substrate, these parameters were critical in facilitating the growth of fruiting bodies. Optimal growth was achieved on substrates maintained at moisture levels from 55% to 65%, containing either 25% or 35% organic matter, under which conditions clusters of fruiting bodies with diameters ranging from 7 to 9 cm and weights between 50 and 120 g were harvested within a period of 30–45 days. The biological efficiency (BE) of fungal growth under these conditions was quantified to range from approximately 12% to 16%.

In 2019, Du, Z. et al. undertook a series of domestication and cultivation experiments utilizing the wild *Fomitopsis betulina* strain YBHB-1, which was harvested from the Changbai Mountain region [8]. The primary substrate selected for these trials was birch sawdust, chosen due to its conducive growth properties, facilitating the successful cultivation of fruiting bodies. The initial phase of the study involved using Potato Dextrose Agar (PDA) as the basal medium, where the mycelium exhibited rapid growth, characterized by its robust and clean structure with minimal aerial development. Following this phase, the researchers prepared a more complex substrate consisting of 80% corn kernels, 10% sawdust, 8% bran, 1% gypsum, and 1% lime, with a moisture content of 60%. Within 18 days, this substrate was fully colonized by the mycelium. For the fruiting experiments, the substrate was adjusted to include 76% birch sawdust, 20% wheat bran, 2% soybean flour, 1% lime, and 1% gypsum, with a moisture range of 60% to 65%. Post-inoculation, mycelial germination occurred within three days, and the substrate was fully colonized between 35 and 40 days. Upon exposure to light, spherical primordia formed on the surface of the cultivation bags, and careful management ensured that mushroom cultivation progressed without disturbing these initial growths. After approximately 15 days, these primordia developed into semi-circular fruiting bodies measuring 8–10 cm in length, 5–8 cm in width, and 1–3 cm in thickness. Upon drying, the fruiting bodies achieved a weight range of 9 to 20 g each. These studies successfully explored the artificial cultivation methods for *F. betulina*, which will provide a reliable reference for the further development and application of this fungus in the future.

## 3. Bioactive Components from *F. betulina*

### 3.1. Terpenes

#### 3.1.1. Triterpenes

Triterpenes represent a diverse class of natural compounds known for their extensive biological activities. To date, more than 22,000 triterpenes have been isolated from natural sources [14]. This group encompasses critical steroidal hormones extensively utilized in medical treatments and natural products like ginsenosides, which are crucial for enhancing human health and improving quality of life [15]. In the fungal kingdom, triterpenes are typically categorized based on their skeletal structures into pentacyclic types, such as lupane, oleanane, ursane, and friedelane. The tetracyclic triterpenes such as lanostane, dammarane, cucurbitane, and ergostane.

**Figure 1:**
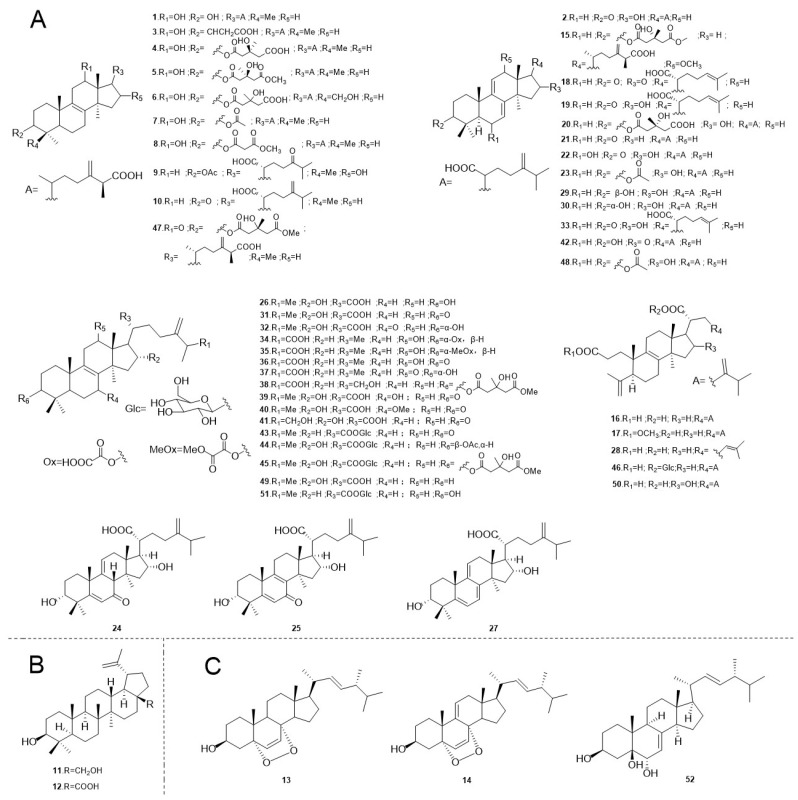
The chemical structures of triterpenoids in *F. betulina.* (**A**) Lanostane-type triterpenoids. (**B**) Lupane-type triterpenoids. (**C**) Ergostane-type triterpenoids.

Specifically in *F. betulina*, research has predominantly identified triterpenes of the lanostane type, which form the largest group of secondary metabolites in this species. Up to now, researchers have documented 47 distinct lanostane-type triterpenoids in *F. betulina*. These tetracyclic triterpenoid compounds derived from lanosterol are generally believed to originate from 2,3(*S*)-epoxycholestane, undergoing cyclization in configurations such as chair–boat–chair or chair–chair–chair. Lanostane-type triterpenoids are a prevalent and characteristic type of triterpenoid found in fungi [14]. Moreover, additional studies have revealed the presence of three types of ergostane triterpenoids and two types of lupane triterpenoids in *F. betulina*, further expanding the chemical diversity of this species (Figure 1).

In 2003, Kamo, T. et al. [16] isolated six distinct lanostane triterpenoid compounds from the fruiting bodies of *F. betulina*. The compounds isolated included two polyporenic acids, identified as polyporenic acid A (**1**) and polyporenic acid C (**2**), along with four derivatives of polyporenic acids: (25*S*)-(+)-12α-hydroxy-3α-malonyloxy-24-methyllanosta-8,24(31)-dien-26-oic acid (**3**), (25*S*,3′*S*)-(+)-12α-hydroxy-3α-(3′-hydroxy-3′-methylglutaryloxy)-24-methyllanosta-8,24(31)-dien-26-oic acid (**4**), (25*S*,3′*S*)-(+)-12α-hydroxy-3α-(3′-hydroxy-4′-methoxycarbonyl-3′-methylbutyryloxy)-24-methyllanosta-8,24(31)-dien-26-oic acid (**5**), and (+)-12α,28-dihydroxy-3α-(3′-hydroxy-3′-methylglutaryloxy)-24-methyllanosta-8,24(31)-dien-26-oic acid (**6**). Among these triterpenoids, all except compound **2** possess the same structural skeleton. This work also represents the first identification of this type of triterpenoid in the fungus. In the following year, Wangun, H.V.K. et al. [17] isolated two previously unreported lanostane-type triterpenes from dried *F. betulina* fruiting bodies, 3α-acetylpolyporenic acid A (**7**) and(25*S*)-(+)-12α-hydroxy-3α-methylcarboxyacetate-24-methyllanosta-8,24(31)-diene-26-oic acid (**8**). These compounds, similar to the lanostane-type triterpenes discovered by Kamo, T. et al. [16], share the same skeletal structure.

Twelve years later, Alresly, Z. et al. [18] re-evaluated the dried fruiting bodies of *F. betulina* and successfully identified a previously unreported lanostane-type triterpenoid, 3β-acetoxy-16 α hydroxy-24-oxo-5α-lanosta-8-ene-21-oic acid (**9**), through silica gel column chromatography and semi-preparative HPLC. They also identified ten known triterpenes. Notably, in addition to the lanostane-type triterpenoid fomefficinic acid (**10**), this study reported the presence of two lupane-type triterpenes, betulin (**11**) and betulinic acid (**12**), as well as two ergostane-type triterpenes, ergosterol peroxide (**13**) and 9,11-dehydroergosterol peroxide (**14**), all of which were reported for the first time in this fungus. In the following year, Tohtahon, Z. et al. [19] isolated five previously unreported lanostane-type triterpenoids from *F. betulina*, namely piptolinic acids A–E (**15**–**19**), as well as five known lanosterol-type triterpenoids: 3-*epi*-(3′-hydroxy-3′-methylglutaryloxyl)-dehydrotumulosic acid (**20**), dehydroeburiconic acid (**21**), 6α-hydroxypolyporenic acid C (**22**), and 3-*epi*-dehydropachymic acid (**23**). These compounds were reported for the first time in this fungus. Interestingly, this study also identified and characterized two types of open-ring lanosterol-type triterpene, **16** and **17**, which had previously been reported as major triterpenes in *Poria cocos* [20].

In 2018, Khalilov, Q. et al. [21] used reverse-phase silica gel column chromatography and semi-preparative HPLC to isolate five previously unreported 24-methyl-lanostane-type triterpenes from dried *F. betulina* fruiting bodies, namely piptolinic acid F–J (**24**–**28**), as well as seven known lanosterol-type triterpenoids. Among them, dehydrotumulosic acid (**29**), 3-*epi*-dehydrotumulosic acid (**30**), 16α-hydroxyeburiconic acid (**31**), 3α,16α-dihydroxy-7-oxo-24-methyllanosta-8,24(31)-dien-21-oic acid (**32**), and 16α-hydroxy-3-oxo-lanosta-7,9(11),24-trien-21-oic acid (**33**) were reported for the first time in *F. betulina*. Compound **29** is a derivative of 3,4-seco-lanosterol.

In a recent study, Sofrenić, I. et al. [22] isolated 13 previously unreported 24-methylene lanostane triterpenes from dried *F. betulina* fruiting bodies, classified as polyporenic acid E–M (**34**–**42**) and fomitoside L–O (**43**–**46**). The investigation further identified sixteen known lanosterol-type triterpenoids and a novel ergostane-type triterpenoid. Among these, several compounds were isolated and characterized for the first time in *F. betulina*, including palustrisoic acid F (**47**), dehydropachymic acid (**48**), pachymic acid (**49**), poricoic acid H (**50**), fomitoside J (**51**), and cerevisterol (**52**). Notably, while most of the isolated compounds were identified as lanosterol-type triterpenoids, Compound **52** was distinguished as an ergostane-type triterpenoid. These compounds not only expand the chemotaxonomic understanding of *F. betulina,* but also highlight the intricate diversity of triterpenoids present within this fungal species.

#### 3.1.2. Sesquiterpenes

**Figure 2:**
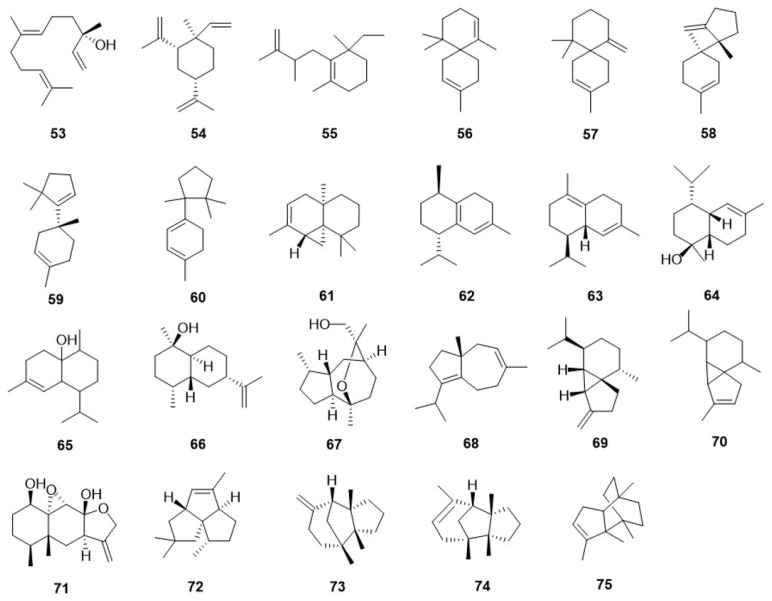
The chemical structures of sesquiterpene in *F. betulina*.

Sesquiterpene are polymers consisting of three isoprene units and exhibit a diverse range of complex skeletal structures [23]. These compounds are notably prevalent in fungi, with over 121 distinct sesquiterpene carbon skeletons identified to date [24]. Twenty-three sesquiterpenes were detected in the fruiting bodies and fermentation broth of *F. betulina* (Figure 2). In 2000, Rösecke, J. et al. [25] detected twenty sesquiterpene compounds from the fresh fruiting bodies of *F. betulina*, including (*R*)-trans-nerolidol (**53**), β-Elemene (**54**), Selina-4,11-diene (**55**), α-Chamigrene (**56**), β-Chamigrene (**57**), β-Bazzanene (**58**), Isobazzanene (**59**), α-Cuprenene (**60**), Thujopsene (**61**), Cadina-1(6),4-diene (**62**), δ-Cadinene (**63**), T-cadinol (**64**), 1-*epi*-cubenol (**65**), (*S*)-(−)-Daucene (**68**), β-Cubebene (**69**), α-Cubebene (**70**), Pentalenene (**72**), (−)-β-Barbatene (**73**), (+)-α-Barbatene (**74**), and Cyclobazzanene (**75**), among which **59**, **68**, **73**, and **74** were reported as fungal components for the first time. In 2015, Sun, C. [26] identified three sesquiterpenes in a rich fermentation broth of *F. betulina*, including one tricyclic sesquiterpene, cryptosphaerolide B (**71**), and two bicyclic sesquiterpenes, rel-(1*S*,4*S*,5*R*,7*R*,10*R*)-10-desmethyl-11-euduemene (**66**) and 10,11-epoxyguaian-13-ol (**67**).

#### 3.1.3. Diterpenes

**Figure 3:**
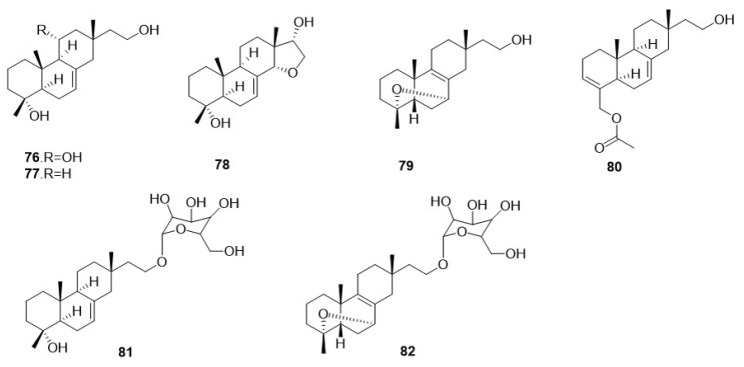
The chemical structures of diterpene in *F. betulina*.

From a rich fermentation broth of *F. betulina*, a total of seven diterpenes have been identified (Figure 3). In 2015, Sun, C. [26] cultured the mycelium of *F. betulina* through liquid fermentation and isolated seven pimarane-type diterpenes from the fermentation broth, specifically identified as pipulinus A–D (**76**–**79**), pipulinus F (**80**), elaeicolasides B (**81**), and pipulinus E (**82**). Compounds **81** and **82** both contain sugar moieties. This study represents the first use of liquid fermentation for the isolation of compounds from *F. betulina*. It not only enriched the methods for extracting natural products from the fungus, but also significantly enhanced the efficiency of compound extraction.

#### 3.1.4. Monoterpenes

**Figure 4:**
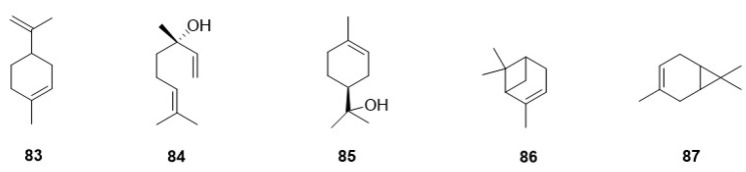
The chemical structures of monoterpene in *F. betulina*.

The chemical investigation of *F. betulina* fruiting bodies has revealed the presence of five distinct monoterpene compounds (Figure 4). In 1995, Rapior, S. et al. [27] detected one monoterpene compound, Limonene (**83**), in the fresh fruiting bodies of *F. betulina*, marking the first known monoterpene from this fungus. Subsequent research by Rösecke, J. et al. [25] in 2000 expanded this list significantly, uncovering four additional monoterpenes: Linalool (**84**), α-Terpineol (**85**), α-Pinene (**86**), and Δ-3-Carene (**87**). These findings deepen the understanding of the volatile constituents of *F. betulina*.

#### 3.1.5. Tetraterpenes

**Figure 5:**
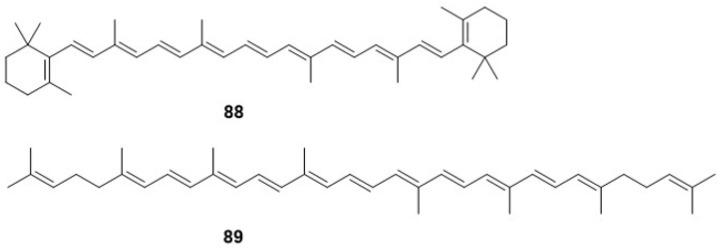
The chemical structures of tetraterpene in *F. betulina*.

In a notable study conducted in 2011, Reis, F.S. et al. [28] analyzed the dried powder of *F. betulina* fruiting bodies and successfully identified two significant tetraterpene compounds (Figure 5). These compounds, β-carotene (**88**) and Lycopene (**89**), known for their potent antioxidant properties, were detected for the first time in this fungal species. This discovery not only enriches our understanding of the phytochemical composition of *F. betulina,* but also suggests potential nutritional and therapeutic applications of these fruiting bodies due to their tetraterpene content.

### 3.2. Phenols

**Figure 6:**
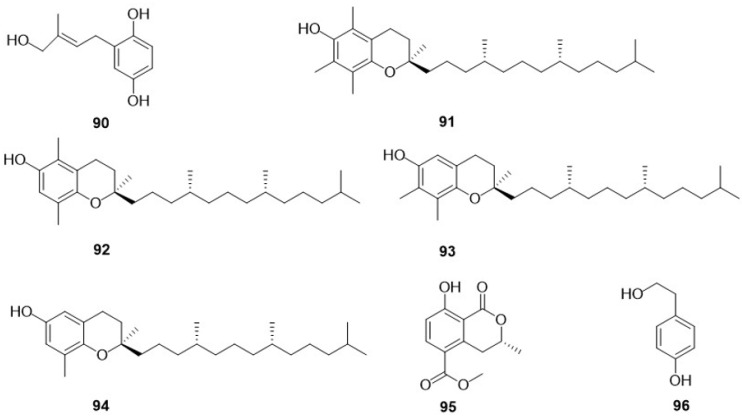
The chemical structures of phenolic compounds in *F. betulina*.

Research into the phenolic content of F. betulina has led to several significant discoveries over the years (Figure 6). In 2002, Kawagishi, H. et al. [29] isolated a novel phenolic compound (*E*)-2-(4-hydroxy-3-methyl-2-butenyl)-hydroquinone (**90**) from the fresh fruiting bodies of *F. betulina*. Further investigations by Reis, F.S. et al. [28] in 2011 expanded the known spectrum of phenolics in this species by detecting four tocopherols, α-tocopherol (**91**), β-tocopherol (**92**), γ-tocopherol (**93**), and δ-tocopherol (**94**), in a dried powder of *F. betulina*. In 2015, Sun, C [26] identified two phenolic compounds, (3*R*)-5-carbomethoxymellein (**95**) and 4-hydroxyphenethyl alcohol (**96**), in a rich fermentation broth of the fungus. These studies have further elucidated the chemical composition of *F. betulina* and enhanced our understanding of this type of fungus.

### 3.3. Others

**Figure 7:**
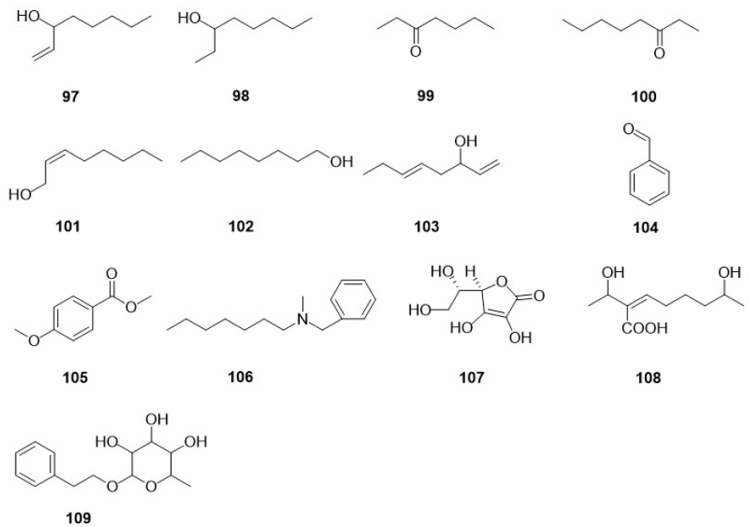
The chemical structures of other compounds in F. betulina.

Over the last few decades, research into the small-molecule compounds present in *F. betulina* has yielded numerous significant discoveries (Figure 7). In 1995, Rapior, S. et al. [27] identified three volatile compounds, 1-octen-3-ol (**97**), 3-octanol (**98**), and 3-heptanone (**99**), from the fresh fruiting bodies of *F. betulina*. In 2000, Rösecke, J. et al. [25] detected six additional volatile small-molecule compounds in the fresh fruiting bodies of *F. betulina*. Among them, 3-Octanone (**100**), (*Z*)-2-octen-1-ol (**101**), 1-Octanol (**102**), and (*Z*)-1,5-octadien-3-ol (**103**) were identified for the first time as small-molecule compounds in *F. betulina*. This research also highlighted the presence of aromatic compounds such as Benzaldehyde (**104**) and Methyl anisate (**105**). In the same year, Schlegel, B. et al. [30] isolated a unique small molecule, piptamine (**106**), from a fermentation broth of *F. betulina*. In 2011, Reis, F.S. et al. [28] detected ascorbic acid (**107**) in a dried powder of *F. betulina*. In 2015, Sun, C [26] isolated two small-molecule compounds, petulinus A (**108**) and 2-phenylethyl-*O*-*β*-rhamnopyranoside (**109**), from a fermentation broth. The chemical structures of these compounds contribute to our understanding of the secondary metabolite diversity and the potential pharmacological uses of *F. betulina*.

## 4. The Biological Activities of Secondary Metabolites from *F. betulina*

**Figure 8:**
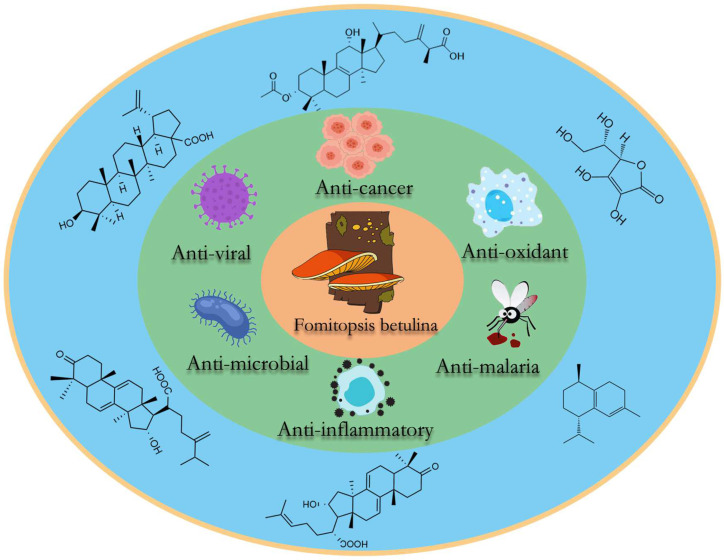
Biological activities of *F. betulina*.

*Fomitopsis betulina* is a large medicinal and edible fungus known for its distinctive biological activities. Its fruiting bodies contain various active compounds that contribute to its rich biological effects (Figure 8). In this study, we review the biological activities of 109 compounds derived from *F. betulina* (Table 1), with a primary focus on its anticancer, anti-inflammatory, and antimicrobial properties.

### 4.1. Anti-Cancer Activity

Currently, a total of 18 terpenoids with proven anti-cancer effects have been identified from the fungus *F. betulina*. The diverse structural features of these compounds contribute to their unique biological activities.

Compounds **2**, **3**, and **7** were evaluated for their anti-proliferative activities against adenocarcinoma cells (Colo 320) using the MTT assay. The results indicated that **2** and **7** exhibited potent inhibitory activities against cancer cells, while **3** showed weaker inhibitory effects. Compound **38**, in combination with doxorubicin, demonstrated synergistic inhibition against Colo 320 cells. Some studies indicated strong synergistic effects when **38** and doxorubicin are used in a ratio of 586.2:1 [31]. Compound **5** demonstrated effective inhibitory effects against the melanoma cell line A-375 and the renal carcinoma cell line 786-O, with Half-Maximal Inhibitory Concentration (IC_50_) values of 42.8 µM and 56.5 µM, respectively. In comparison, the positive control doxorubicin exhibited IC_50_ values of 1.3 µM and 42.8 µM against these cell lines [21].

Compound **13** was evaluated for its cytotoxic effects using the MTT assay against three prostate cancer cell lines (DU145, PC3, and M2182), as well as lung cancer cells (A549), colon cancer cells (HCT116), ovarian cancer cells (SKOV3), and normal prostate epithelial cells (RWPE1). The results demonstrated that **13** exerted strong cytotoxic effects on all the tested cell lines, showing a concentration-dependent cytotoxic effect [32].Additionally, **13** exhibited significant inhibitory effects against hepatocellular carcinoma cells (Hep3B), with an IC_50_ of 8.3 µM. Under the same conditions, compound **14** demonstrated a cytotoxic effect against Hep3B cells, with an IC_50_ of 7.1 µM. These study results highlight the potent proliferation inhibition activity of both **13** and **14** against Hep3B cells [33].

Compound **15** has been shown to exhibit cytotoxic effects against the human acute promyelocytic leukemia cell line HL-60 and the human acute monocytic leukemia cell line THP-1 comparable to those of fluorouracil [19]. Compounds **5**, **30**, **43**, **45**, **48**, and **49** were tested for cytotoxicity against HL60 cells. The study revealed that **5**, **30**, **48**, and **49** not only exhibited cytotoxic activity against HL60 cells, but also showed selectivity towards healthy MRC-5 cells, with IC_50_ values of 19.2 µM, 19.9 µM, 10.9 µM, and 11 µM, respectively. Compounds **43** and **45** demonstrated IC_50_ values of 15.8 µM and 23.7 µM against HL60 cells [22]. Compound **54** has been demonstrated to exhibit cytotoxic effects against HL-60 and the human chronic myelogenous leukemia cell line K-562, with IC_50_ values of 5.6 µM and 16.5 µM, respectively. Moreover, this natural product shows promising inhibitory effects against cervical cancer, as well as various solid tumors, including lung, liver, and brain cancers, with minimal side effects [34]. These activity assessments highlight the potential of various natural products from *F. betulina* to serve as novel anti-leukemia drugs.

Compounds **16** and **18** have been shown to inhibit steroid sulfatase (STS), which is involved in increasing the free steroid levels in tumor cells. Compounds **16** and **18** exhibit inhibition effects on STS at 72% and 74% of irosustat, respectively, highlighting the significant potential of natural products as STS inhibitors [35].

In studies investigating the cytotoxic effects on lung cancer cells (A549) and prostate cancer cells (DU145), compounds **23** and **30** were found effective, with IC_50_ values of 24.0 µM and 56.8 µM for A549, and 25.3 µM and 418.6 µM for DU145, respectively [36]. In studies assessing anti-cancer activity against colorectal cancer cells (HT-29), compound **89** demonstrated significant anti-proliferative effects at low concentrations. Its IC_50_ value in the HT-29 cells within 24 h was determined to be 4.382 µM. This research also validated **89** for its anti-proliferative, apoptotic, and genotoxic effects on HT-29 colorectal cancer cells [37]. Compound **94** has been demonstrated to attenuate growth factor-induced AKT activation in prostate cancer cells, thereby inhibiting proliferation and inducing apoptosis [38].

### 4.2. Anti-Inflammatory Activity

Currently, a total of 16 compounds with anti-inflammatory properties have been identified from *F. betulina*.

Compounds **1**–**6** have been shown to inhibit TPA-induced inflammation in mouse ear models. At a concentration of 400 nmol/L, these compounds exhibited swelling inhibition rates of 64%, 49%, 65%, 76%, 86%, and 75%, respectively. Compounds **1** and **3**–**6** demonstrated stronger inhibitory activities compared to those of glycyrrhetic acid and Indomethacin [16].

Using Indomethacin as a positive control, compounds **2**, **5**, **7**, and **8** were evaluated for their anti-inflammatory activity using the 3α-HSD method. Their IC_50_ values were determined to be 17.5 µM, 5.5 µM, 8.5 µM, and 4.0 µM, respectively. Compounds **2**, **5**, and **7** exhibited anti-inflammatory activities comparable to those of Indomethacin, while **8** demonstrated more anti-inflammatory activity than Indomethacin [17]. Compound **11** has been demonstrated to significantly reduce the production of nitric oxide (NO). At a concentration of 10 µM, **11** decreased NO production by more than 50%. Compound **12**, on the other hand, effectively reduced the production of interleukin-6 (IL-6), a key pro-inflammatory cytokine in innate and adaptive immunity. Additionally, **11** and **12** exhibited the moderate inhibition of activated macrophage iNOS expression, with reductions of 37% and 42%, respectively [39].

Compound **21** exhibits inhibitory effects on TPA-induced inflammation in mouse ear models. The measurement of ear thickness after 6 h of TPA induction showed that at a dose of 1 µg/ear, **21′**s inhibitory effect is comparable to that of Hydrocortisone [40]. Compound **29** has been shown to significantly reduce the ear swelling induced by ethyl phenylpropiolate in mice. At a dose of 0.5 mg/ear, it exhibited approximately 50% inhibition of swelling. Furthermore, in mice with paw edema induced by PLA2, **29** showed the inhibition of edema close to 60% at a concentration of 50 mg/kg after 60 min [41].

Compound **52** has been demonstrated to inhibit the production of nitric oxide (NO) and prostaglandin E2 (PGE2) induced by endotoxin. It also reduces the expression levels of the pro-inflammatory cytokines TNF-α, IL-6, and IL-1β. Additionally, **52** inhibits the transcriptional activation of nuclear factor-kappa B (NF-κB) and the mitogen-activated protein kinase (MAPK) signaling pathways. It further inhibits AP-1 transcriptional activation and the phosphorylation of c-Fos. Moreover, **52** induces the nuclear translocation of nuclear factor erythroid 2-related factor 2 (Nrf2) by downregulating Kelch-like ECH-associated protein 1 (KeAP-1) and upregulating hemeoxygenase-1 (HO-1) expression. These findings suggest that **52** could serve as a natural therapeutic agent for inflammatory diseases by targeting the MAPK, NF-κB, AP-1, and NRF2-mediated HO-1 signaling pathways [42].

Compounds **88** and **89** have been shown to upregulate Hmox1 mRNA expression and inhibit the expression of COX_2_, NOS_2_, and TNF-α genes induced by lipopolysaccharide (LPS) stimulation. Research suggests their anti-inflammatory activity may be associated with their ability to scavenge reactive oxygen species (ROS). Compound **107** is widely recognized for its multifaceted anti-inflammatory properties. While specific data on its impact on pro-inflammatory cytokines like IL-6 are lacking, when used in combination with other compounds, such as α-tocopherol, β-carotene, and 25-hydroxyvitamin D, **107** significantly downregulates IL-6 and interferon-γ, among other pro-inflammatory molecules, and variably affects IL-4 levels in human plasma [43,44].

### 4.3. Antimicrobial Activity

Currently, a total of five compounds with antibacterial activity have been isolated from *F. betulina*.

Compound **2** exhibited significant antibacterial activity against *Mycobacterium phlei* in antimicrobial susceptibility tests involving *Staphylococcus aureus*, *Bacillus coli*, and *Mycobacterium phlei*. At a concentration of 1 mg/mL, **2** continued to inhibit the growth of *Mycobacterium phlei* for up to 72 h even after being diluted 6.4 × 10^5^ times. Inhibition persisted for as long as 7 days, whereas its inhibitory effects on *Staphylococcus aureus* and *Bacillus coli* were comparatively weaker [45].

When using the agar diffusion method to assess the antibacterial effects of compounds **1**, **2**, **4**, **8**, **9**, **10**, **11**, **12**, **13**, and **14**, compound **9** exhibited notable antibacterial activity against Gram-positive bacteria. It demonstrated minimal inhibitory concentrations (MIC) of 98 μg/mL against *Staphylococcus aureus* and approximately 200 μg/mL against *Bacillus subtilis*. However, its inhibitory effects against the Gram-negative strains were weaker. Compounds **2**, **4**, **8**, **10**, **13**, and **14** showed weaker inhibitory effects against *Bacillus subtilis* and *Staphylococcus aureus* [18]. Compound **64** has been demonstrated to possess potent bacteriolytic effects. It exhibits a minimum inhibitory concentration (MIC) of 24 µg/mL against *Staphylococcus aureus* and a minimum fungicidal concentration (MFC) of 2.3 µg/mL against Trichophyton mentagrophytes. Its bactericidal action primarily involves dissolving bacterial cell membranes, leading to cell death [46].

In research exploring secondary metabolites for their efficacy against plant pathogens, compound **65** has been shown to be effective. It exhibited a 76% inhibition rate against Rhizoctonia solani and a 92% inhibition rate against *Choanephora cucurbitarum*. The study results confirm the potential of **65** as a candidate for controlling fungal diseases in plants [47]. In studies of the antibacterial activity of compound **106**, it exhibited antibacterial activity against a range of Gram-positive bacteria, yeast, and fungi. The MIC values for *Staphylococcus aureus* SG 511 and *Enterococcus faecalis* 1528 were 0.78 µg/mL and 1.56 µg/mL, respectively [30].

**Table 1:** Compounds isolated from *F. betulina* and their activities.

Number	Compound Names	Biological Activities	Materials	References
Triterpenes				
**1**	polyporenic acids A	P-gp efflux pump inhibitory Anti-inflammatory	fruit body	[16,31]
**2**	polyporenic acids C	Anti-cancer P-gp efflux pump inhibitory Antibacterial Anti-inflammatory	fruit body	[16,17,31,45]
**3**	(25*S*)-(+)-12α-hydroxy-3α-malonyloxy-24-methyllanosta-8,24(31)-dien-26-oic acid	Anti-cancer Anti-inflammatory	fruit body	[16,31]
**4**	(25*S*,3′*S*)-(+)-12α-hydroxy-3α-(3′-hydroxy-3′-methylglutaryloxy)-24-methyllanosta-8,24(31)-dien-26-oic acid	Anti-inflammatory	fruit body	[16]
**5**	(25*S*,3′*S*)-(+)-12α-hydroxy-3α-(3′-hydroxy-4′-methoxycarbonyl-3′-methylbutyryloxy)-24-methyllanosta-8,24(31)-dien-26-oic acid	Anti-cancer Anti-inflammatory	fruit body	[16,17,21,22]
**6**	(+)-12α,28-dihydroxy-3α-(3′-hydroxy-3′-methylglutaryloxy)-24-methyllanosta-8,24(31)-dien-26-oic acid	Anti-inflammatory	fruit body	[16]
**7**	3α-acetylpolyporenic acid A	Anti-cancer P-gp efflux pump inhibitory Anti-inflammatory	fruit body	[17,31]
**8**	(25*S*)-(+)-12α-hydroxy-3α-methylcarboxyacetate-24-methyllanosta-8,24(31)-diene-26-oic acid	P-gp efflux pump inhibitory Anti-inflammatory	fruit body	[17,31]
**9**	3β-acetoxy-16 α hydroxyl-24-oxo-5*α*-lanosta-8- ene-21-oic acid	Antibacterial	fruit body	[18]
**10**	fomefficinic acid	-	fruit body	[18]
**11**	betulin	Anti-inflammatory	fruit body	[18,39]
**12**	betulinic acid	Anti-inflammatory Antiviral Anti-malarial	fruit body	[18,39,48,49]
**13**	ergosterol peroxide	Anti-parasitic Anti-cancer	fruit body	[18,32,33,50]
**14**	9,11-dehydroergosterol peroxide	Anti-cancer	fruit body	[18,33]
**15**	piptolinic acids A	Anti-cancer	fruit body	[19]
**16**	piptolinic acids B	Anti-cancer	fruit body	[19,35]
**17**	piptolinic acids C	-	fruit body	[19]
**18**	piptolinic acids D	Anti-cancer	fruit body	[19,35]
**19**	piptolinic acids E	-	fruit body	[19]
**20**	3-*epi*-(3′-hydroxy-3′-methylglutaryloxyl)-dehydrotumulosic acid	-	fruit body	[19]
**21**	dehydroeburiconic acid	Anti-inflammatory	fruit body	[19,40]
**22**	6α-hydroxypolyporenic acid C	acetylcholine receptor inhibitors	fruit body	[19,51]
**23**	3-epidehydropachymic acid	Anti-cancer	fruit body	[19,36]
**24**	piptolinic acid F	-	fruit body	[21]
**25**	piptolinic acid G	-	fruit body	[21]
**26**	piptolinic acid H	-	fruit body	[21]
**27**	piptolinic acid I	-	fruit body	[21]
**28**	piptolinic acid J	-	fruit body	[21]
**29**	dehydrotumulosic acid	Anti-inflammatory	fruit body	[21,41]
**30**	3-*epi*-dehydrotumulosic acid	Anti-cancer	fruit body	[21,22,36]
**31**	16α-hydroxyeburiconic acid	-	fruit body	[21]
**32**	3α,16α-dihydroxy-7-oxo-24-methyllanosta-8,24(31)-dien-21-oic acid	-	fruit body	[21]
**33**	16α-hydroxy-3-oxo-lanosta-7,9(11),24-trien-21-oic acid	Anti-inflammatory	fruit body	[21,52]
**34**	polyporenic acid E	-	fruit body	[22]
**35**	polyporenic acid F	-	fruit body	[22]
**36**	polyporenic acid G	-	fruit body	[22]
**37**	polyporenic acid H	Strong synergistic interaction with Doxorubicin	fruit body	[31]
**38**	polyporenic acid I	-	fruit body	[22]
**39**	polyporenic acid J	-	fruit body	[22]
**40**	polyporenic acid K	-	fruit body	[22]
**41**	polyporenic acid L	-	fruit body	[22]
**42**	polyporenic acid M	-	fruit body	[22]
**43**	fomitoside L	Anti-cancer	fruit body	[22]
**44**	fomitoside M	-	fruit body	[22]
**45**	fomitoside N	Anti-cancer	fruit body	[22]
**46**	fomitoside O	-	fruit body	[22]
**47**	palustrisoic acid F	-	fruit body	[22]
**48**	dehydropachymic acid	Anti-cancer	fruit body	[22]
**49**	pachymic acid	Anti-cancer	fruit body	[22]
**50**	poricoic acid H	-	fruit body	[22]
**51**	fomitoside J	-	fruit body	[22]
**52**	cerevisterol	Anti-inflammatory	fruit body	[42]
Sesquiterpenes				
**53**	(*R*)-trans-nerolidol	-	fruit body	[25]
**54**	β-Elemene	Anti-cancer	fruit body	[34]
**55**	Selina-4,11-diene	-	fruit body	[25]
**56**	α-Chamigrene	-	fruit body	[25]
**57**	β-Chamigrene	-	fruit body	[25]
**58**	β-Bazzanene	-	fruit body	[25]
**59**	Isobazzanene	-	fruit body	[25]
**60**	α-Cuprenene	-	fruit body	[25]
**61**	Thujopsene	Anti-fungal	fruit body	[25,53]
**62**	Cadina-1(6),4-diene	-	fruit body	[25]
**63**	δ-Cadinene	Anti-malarial	fruit body	[25,54]
**64**	T-cadinol	Antibacterial	fruit body	[25,46]
**65**	1-*epi*-cubenol	Antibacterial	fruit body	[25,47]
**66**	rel-(1*S*,4*S*,5*R*,7*R*,10*R*)-10-desmethyl-11-euduemene	-	fermentation broth	[26]
**67**	10,11-epoxyguaian-13-ol	-	fermentation broth	[26]
**68**	(*S*)-(−)-Daucene	-	fruit body	[25]
**69**	β-Cubebene	-	fruit body	[25]
**70**	α-Cubebene	-	fruit body	[25]
**71**	cryptosphaerolide B	-	fermentation broth	[26]
**72**	Pentalenene	-	fruit body	[25]
**73**	(−)-β-Barbatene	-	fruit body	[25]
**74**	(+)-α-Barbatene	-	fruit body	[25]
**75**	Cyclobazzanene	-	fruit body	[25]
Diterpenes				
**76**	pipulinus A	-	fruit body	[26]
**77**	pipulinus B	-	fermentation broth	[26]
**78**	pipulinus C	-	fermentation broth	[26]
**79**	pipulinus D	-	fermentation broth	[26]
**80**	pipulinus F	-	fermentation broth	[26]
**81**	elaeicolasides B	-	fermentation broth	[26]
**82**	pipulinus E	-	fermentation broth	[26]
Monoterpenes				
**83**	Limonene	-	fruit body	[27]
**84**	Linalool	-	fruit body	[25]
**85**	α-Terpineol	-	fruit body	[25]
**86**	α-Pinene	-	fruit body	[25]
**87**	Δ-3-Carene	-	fruit body	[25]
Tetraterpenes				
**88**	β-carotene	Anti-inflammatory	fruit body	[28,55]
**89**	Lycopene	Antioxidant Anti-cancer Anti-inflammatory	fruit body	[28,37,55,56]
Phenols				
**90**	(*E*)-2-(4-hydroxy-3-methyl-2-butenyl)-hydroquinone	metalloprotein inhibitor	fruit body	[29]
**91**	α-tocopherol	acetylcholinesterase inhibitors	fruit body	[28,57]
**92**	β-tocopherol	-	fruit body	[28]
**93**	γ-tocopherol	Antioxidant	fruit body	[28,58]
**94**	δ-tocopherol	Inhibit alpha-glucosidase Anti-cancer	fruit body	[28,38,59]
**95**	(3*R*)-5-carbomethoxymellein	-	fermentation broth	[26]
**96**	4-hydroxyphenethyl alcohol	Cytokinins	fermentation broth	[26,60]
Others				
**97**	1-octen-3-ol	-	fruit body	[27]
**98**	3-octanol	-	fruit body	[27]
**99**	3-heptanone	-	fruit body	[27]
**100**	3-Octanone	-	fruit body	[25]
**101**	(*Z*)-2-octen-1-ol	-	fruit body	[25]
**102**	1-Octanol	-	fruit body	[25]
**103**	(*Z*)-1,5-octadien-3-ol	-	fruit body	[25]
**104**	Benzaldehyde	-	fruit body	[25]
**105**	Methyl anisate	-	fruit body	[25]
**106**	piptamine	Antibacterial	fruit body	[30]
**107**	Ascorbic acid	Antioxidant Anti-inflammatory	fruit body	[28,61]
**108**	petulinus A	-	fermentation broth	[26]
**109**	2-phenylethyl-*O-β*-rhamnopyranoside	-	fermentation broth	[26]

## 5. Sesquiterpene Biosynthesis Analysis of *F. betulina*

*F. betulina* harbors a diverse array of terpenoid and sesquiterpenoid compounds recognized for their unique physiological and medicinal properties. Despite substantial research focused on isolating and identifying these bioactive compounds, their biosynthetic pathways remain underexplored. The advent of genome sequencing technology has revealed biosynthetic gene clusters in various fungi, including *Flammulina velutipes* [62], *Hericium erinaceus* [63], *Inonotus obliquus* [64], and *Inonotus hispidus* [65], with their secondary metabolite genes progressively elucidated. However, as a large fungus capable of producing multiple active compounds, *F. betulina* has not received adequate attention. We have downloaded the genome of *F. betulina* strain CIRM-BRFM 1772 from the NCBI, with a genome size of 42.86 Mb [66]. Utilizing the antiSMASH tool, we predicted the terpene synthases implicated in the biosynthesis of sesquiterpenes within *F. betulina*. From the annotated genome data available from the NCBI, we identified and annotated 20 sesquiterpene synthase genes. After the initial screening and the removal of two squalene synthase genes, we narrowed the candidates down to 18. By employing reference terpene synthases from *Omphalotus olearius* VT-653.13 [67], *Stereum hirsutum* FP-91666 SS1 [68], and *Coprinopsis cinerea* 9/55 [69] as standards to classify the sesquiterpene synthase types in *F. betulina*, we conducted the analysis of the sesquiterpene synthase types present in this fungus.

**Figure 9:**
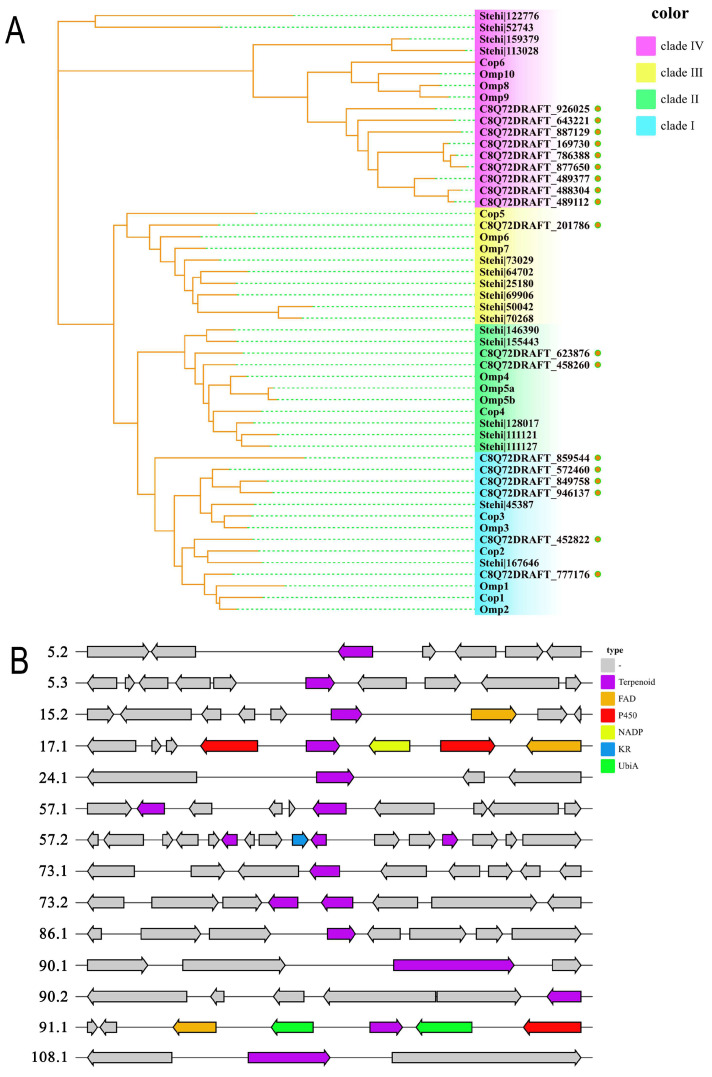
The phylogenetic analysis and gene clusters of the predicted sesquiterpene synthase homologs. (**A**) displays the neighbor-joining phylogenetic tree of sesquiterpene synthase homologous proteins, generated using MEGA (version 10.0) software. To classify the 18 sesquiterpene synthases in *F. betulina*, selected 33 sesquiterpene synthases from *O. olearius*, *S. hirsutum*, and *C. cinereus* as references and constructed a phylogenetic tree of 51 sequences using 1000 bootstrap replicates in MEGA. (**B**) shows the predicted sesquiterpene synthase gene clusters in *F. betulina*.

Fungal sesquiterpene synthases are typically classified into four branches. Clade I: Involves the formation of a 10-membered ring carbocation intermediate and a E,E-germacradienyl cation via the 1,10-cyclization of (2E,6E)-FPP. Clade II: Generates an E-germacradienyl cation through the 1,10-cyclization of (3R)-nerolidyl diphosphate. Clade III: Leads to the creation of an 11-membered ring carbocation intermediate, namely, trans-caryophyllene cation, via 1,11-cyclization. Clade IV: Forms a six-membered ring carbocation intermediate, (6R)-β-bisabolol cation, through the 1,6-cyclization of (3R)-NPP [70]. As shown in Figure 9A, through the analysis of the amino acid sequences and evolutionary relationships of 18 terpene synthases in *F. betulina*, we found that these sesquiterpene synthases are primarily distributed among class I and class IV, with six and nine representatives, respectively. The remaining sequences are distributed in class II and class III, with two and one representatives, respectively. In recent years, heterologous expression has become an important tool for elucidating gene functions and efficiently producing valuable natural products [71]. To facilitate subsequent research on sesquiterpene synthases in *F. betulina*, we predicted and annotated the sesquiterpene synthase gene clusters in *F. betulina*, as shown in Figure 9B. The biosynthetic gene clusters 57.1, 57.2, and 73.3 each contain two or more sesquiterpene synthase genes. Apart from the sesquiterpene synthase, cluster 17.1 contains two P450 monooxygenases, one NADP-dependent dehydrogenase, and one FAD monooxygenase. In cluster 91.1, besides the sesquiterpene synthase, there are also two Ubia-type isoprenyl transferases, one P450 monooxygenase, and one FAD monooxygenase. In the future, by using the heterologous expression of these genes or gene clusters, we can uncover the mechanisms of their synergistic effects and pave the way for synthesizing complex sesquiterpenes that are rare or previously unobserved in nature.

## 6. Conclusions

In this review, we primarily summarized 109 secondary metabolites and various small molecules discovered from the *F. betulina* to date, along with the extraction materials. The extraction materials encompassed both the fruiting bodies and mycelial fermentation broth. We also outline the pharmacological activities of these compounds, including anti-cancer, anti-inflammatory, antimicrobial, and antioxidant properties. Mushrooms constitute a natural reservoir of secondary metabolites. However, many bioactive natural products are produced in insufficient quantities by their native hosts. In recent years, the rapid advancement of genome sequencing and the emergence of CRISPR-Cas9 gene editing technology have enabled the heterologous expression of genes for bioactive natural products. Consequently, the significance of developing biosynthesis pathways for natural products is self-evident. Simultaneously pursuing the heterologous synthesis of target natural products and deciphering biosynthetic gene clusters not only deepens our understanding of secondary metabolite pathways in mushrooms, but also paves the way for the development of new drugs and other potential applications.

## Data Availability

All relevant data generated or analyzed during this study are included in this manuscript.
